# Continuous Valorization of Carbon Dioxide into the Fine Chemical Ectoine by Hydrogenovibrio marinus: A New Strategy for Pharmaceutical Production

**DOI:** 10.1021/acs.est.4c12259

**Published:** 2025-03-05

**Authors:** E. Huang-Lin, R. Lebrero, S. Cantera

**Affiliations:** † Department of Chemical Engineering and Environmental Technology, 16782 University of Valladolid, Dr. Mergelina s/n., 47011 Valladolid, Spain; ‡ Institute of Sustainable Processes, University of Valladolid, Dr. Mergelina s/n., 47011 Valladolid, Spain

**Keywords:** CO_2_ valorization, halophiles, extremolytes, hydrogen-oxidizing bacteria, Knallgas bacteria

## Abstract

Current challenges in biopharmaceutical manufacturing, such as ectoine production, include high operational costs and limited availability. Transitioning to processes that valorize renewable carbon sources like CO_2_ into ectoine can make production more sustainable and accessible to the economy and society. However, cell platforms that produce ectoine with CO_2_ still require bioprocess optimization and resilient microorganisms able to continuously maintain high ectoine yields and CO_2_ removals. A comprehensive screening of cultivation and operational strategies was conducted in six stirred-tank gas bioreactors using the strain Hydrogenovibrio marinus, a halophilic, fast-growing, hydrogenotrophic bacterium with low nutrient requirements. Gas residence times of 120 min at gas ratios of 10:40:50 CO_2_:H_2_:air (% v/v) and dilution rates of 0.25 d^–1^ boosted ectoine production and biomass growth during long-term operation. Under these conditions, ectoine productivity reached 5.0 ± 0.3 g m^–3^ d^–1^, with maximum specific ectoine contents of 134.0 ± 6.3 mg_Ect_ g_biomass_^–1^, achieving yields similar to heterotrophic strains. This study demonstrates for the first time the feasibility of integrating ectoine production with continuous CO_2_ abatement using H_2_ as a clean and hazard-free energy source, which marks a significant advancement in sustainable ectoine manufacturing and CO_2_ circularity.

## Graphical abstract

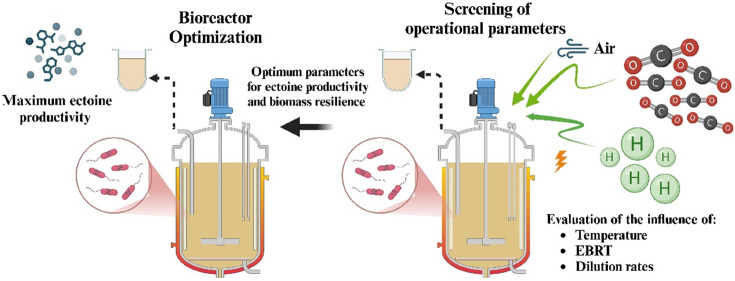

## 1 Introduction

The defossilization of the chemical industry requires the development of new processes where renewable carbon sources, such as carbon dioxide (CO_2_), are used. The production of valuable biochemicals including antimicrobials (halocin, quinolones, bacterioruberin, carotenoids)^1^; cancer chemo-preventive agents (carotenoids, biosurfactants)^2,3^ ; preventive compounds for cardiovascular and degenerative diseases (natural coenzymes)^4^; anti-inflammatory and antiaging osmo-protectants (ectoine, hydroxyectoine),^5,6^ plays a crucial role in addressing essential human needs while advancing a green industry. However, current production processes for these valuable chemicals often rely on chemical synthesis or extraction from animal and plant tissues.^7^ In the limited cases where biotechnological approaches are employed, they typically depend on costly carbon sources (e.g., glucose, peptone, lipids), which compete with the food industry and involve energy-intensive processes, significant freshwater usage, inefficient batch fermentation, and complex purification procedures.^8^ Moreover, the low availability of these biobased products is largely due to the implementation of a narrow number of model organisms and the limited portfolio of biobased products synthesized.^9^ To overcome these challenges, the adoption of a “next-generation industrial biotechnology” is essential. This approach focuses on advancing green bioprocesses, with more sustainable biobased products, a broader number of chemicals produced, a reduction in the use of fresh water, and novel resistant microorganisms as catalysts.^10^

Organisms that thrive in high-salinity environments present a promising option for the development of green chemical factories.^11,12^ These organisms exhibit versatile metabolism, capable of utilizing a wide range of carbon and energy sources. They do not require fresh water, promote contamination-free environments, and can produce various interesting chemicals to protect themselves against osmotic stress, such as enzymes, biopolymers, extracellular polymeric substances and compatible solutes as macromolecules stabilizers.^11,13–15^ Within this last group, an interesting osmolyte is ectoine ((*S*)-2-Methyl-1,4,5,6-tetrahydroprimidine-4-carboxylic acid). Ectoine is classified as a pivotal heterocyclic amino acid with broad applicability in research and industrial fields, recognized for its therapeutic benefits such as treating atopic dermatitis, allergic rhinitis, pulmonary inflammation, Alzheimer’s disease, or intestinal disorders. Additionally, it functions as a cryoprotectant for cells and tissues and as a natural preservative in the food industry.^16^ These distinctive characteristics of ectoine can enhance and expand the potential uses of biobased chemicals. This compound is typically synthesized by halophilic or halotolerant microorganisms under salinity stress conditions and it assumes a vital role in regulating elevated turgor pressures and maintaining osmotic equilibrium within the producing cells, all while ensuring uninterrupted cellular metabolic functions.^17^ Currently, ectoine has a retail market value of 400–1000 $/kg-ectoine, a global market size of USD 0.07 billion in 2023, and a projected compound annual growth rate (CAGR) of 6.7%.^15,16,18^ The commercial production of ectoine primarily utilizes fermentation processes with glucose as substrate and a super leaky mutant of the halophilic microorganism Halomonas elongata, achieving titers of 7.4 g L^–1^.^15^ However, this production process mainly relies on sugars or rich carbon sources under extremely high salinity conditions (15–20% NaCl), which diminishes the profitability of the product and directly competes with the food market.^16^

In this regard, the pursuit of innovative bioproduction systems that utilize sustainable feedstocks, C_1_ waste compounds, and renewable energy sources for ectoine production holds great promise, aligning with the goals outlined by the European pharmaceutical strategy, which aims to minimize waste production, reduce emissions, and lower the resource intensity of manufacturing processes.^19^ Nevertheless, there remains a notable gap in comprehensive research focused on achieving these goals.^20^ For instance, various halotolerant, aerobic methane-consuming bacteria have demonstrated the ability to produce ectoine, with the strain Methylomicrobium alkaliphilum achieving 37 mg_Ect_ g_biomass_^–1^ in stirred tank reactors and 109 mg_Ect_ g_biomass_^–1^ in high-mass-transfer bioreactors using methane as sole carbon and energy source.^21,22^ Research suggests that using methanotrophic consortia with biogas as feedstock enhances resilience, achieving ectoine concentrations of 94 mg_Ect_ g_biomass_^–1^.^23^ Nevertheless, the use of biogas as feedstock still faces challenges, including the adaptation of methanotrophs to large-scale production due to methane mass transfer limitations, gas feed quality, the efficiency of microbial catalysts and the significant release of CO_2_ emissions, which diminishes the sustainability of the process.^24^ To address these rate-limiting factors, researchers are exploring the use of different C_1_ gases, like CO_2_, to enhance the sustainability of this bioconversion platform. CO_2_ is present in low concentrations in waste gas streams from sources such as natural gas-fired power plants (5–10% CO_2_), or syngas derived from biomass and coal gasification (10–25% CO_2_).^25^ In this context, these streams could be integrated into this bioconversion process, thereby improving the sustainability of the technology. Additionally, this technology could also be applied to biogas upgrading processes, which typically contain CO_2_ concentrations ranging from 5 to 30%.^26^

Recent studies have shown the potential of unexplored hydrogen-oxidizing strains to synthesize ectoine using CO_2_ as sole carbon source and H_2_ as energy donor at relatively low salt concentrations.^27^ Among these strains, Hydrogenovibrio marinus DSM 11271 exhibited doubling times inferior to 24 h (7.9 ± 0.9 h) and a specific ectoine production of 72.2 ± 10.7 mg_Ect_ g_biomass_^–1^ when grown at 6% NaCl.^27^ These encouraging findings underscore the potential of a novel research platform focused on the sustainable production of pharmaceuticals from CO_2_. Nevertheless, this technology still requires implementation and optimization in gas fermentation to enhance ectoine productivity and improve gas–liquid mass transfer. Therefore, optimizing cultivation conditions to increase ectoine accumulation, along with developing operational strategies for continuous ectoine production in bioreactors, are crucial steps to ensure the technical and economic viability of the process.

In this context, the primary aim of this research was to demonstrate the continuous production of ectoine using CO_2_ as the sole carbon source in stirred tank bioreactors (STR) using the strain H. marinus. First, we systematically evaluated the influence of different operational parameters, including the gas residence time, temperature, and dilution rates. Following this preliminary assessment, we optimized the operational strategy to enhance performance and process resilience in order to achieve higher biomass contents and ectoine productivities.

## 2 Materials and Methods

### 2.1 Chemicals and Mineral Salt Medium

Ammonium Mineral Salt medium (AMS) supplemented with 6% of NaCl was used for the growth of the strain. The medium was composed of (g L^–1^): MgSO_4_·7H_2_O–1.0, CaCl_2_·2H_2_O–0.11, NH_4_Cl_2_–0.5, KNO_3_–1.0, K_2_HPO_4_–1.0. Medium was supplemented with trace elements (mg L^–1^): CuCl_2_–0.01, FeCl_2_–0.9, ZnCl_2_–0.06, NiCl_2_–0.01, CoCl_2_–0.06, Na_2_MoO_4_–0.03, MnCl_2_–0.06, H_3_BO_3_–0.06, Na_2_SeO_3_–0.4, Na_2_WO_4_–0.01). 60 g L^–1^ of NaCl were added during AMS preparation. The medium was autoclaved at 1.5 atm at 121 °C for 20 min. The pH of the medium was adjusted to a final pH of 7.0 using 3 M NaOH stock solution after autoclavation. The vitamins solution (mg L^–1^): biotin–0.02, nicotinamid–0.2, *p*-aminobenzoic acid–0.1, thiamin–0.2, pantothenic acid–0.1, pyridoxamine–0.5, cyanocobalamine–0.1, riboflavine–0.1 was added via filtration through sterilized Millipore filters of 0.22 μm pore-size after autoclavation from a stock solution.

### 2.2 Microorganism and Inoculum Preparation

Hydrogenovibrio marinus DSM 11271, an obligately chemolithoautotrophic hydrogen-oxidizing strain capable to synthesize ectoine,^27^ was acquired as an actively growing culture from DSMZ (Leibniz-Institut, Germany). An aliquot of 1 mL of H. marinus stock liquid culture was inoculated in triplicate in 120 mL glass serum bottles containing 50 mL of AMS with 3% NaCl (30 g L^–1^), which is the recommended NaCl concentration for H. marinus growth according to DSMZ guidelines. The bottles were closed with gastight butyl septa and aluminum caps and CO_2_ and H_2_ were then injected to the headspace in order to reach an initial concentration of 10% CO_2_, 40% H_2_, 50% Air (v/v). The inoculum was grown at 37 °C under orbital agitation at 150 rpm. When the biomass reached the exponential growth phase, the inoculum was transferred to triplicate 120 mL glass serum bottles containing fresh AMS medium supplemented with 6% NaCl (60 g L^–1^), prepared in the same manner. This transfer was repeated three consecutive times to ensure complete adaptation of the biomass to 6% NaCl. Once H. marinus reached active growth and biomass concentrations of 50 mg L^–1^, it was used as inoculum for the continuous bioreactors.

### 2.3 Experimental Setup

The experimental setup consisted of two experimental assays. In the first assay, various operational parameters: Empty Bed Residence Time (EBRT), temperature, and dilution rate were assessed in order to evaluate their influence on bacterial growth, ectoine production, and process resilience. Based on the results of this initial screening, the second experimental assay was carried out using the optimal parameters identified to promote maximum ectoine productivity and long-term operational performance.

All the experimental assays were carried out in a sterile 1-L jacketed stirred tank reactor (STR) (Afora S.A, Spain) equipped with a magnetic stirrer (Agimatic S, JP Selecta, Spain, 200 rpm) located at the bottom of the reactor to ensure an adequate mixing (Scheme 1). The STR was filled with 500 mL of sterile AMS 6% NaCl, and 500 mL of the inoculum prepared (as outlined in section 2.1).

**Scheme 1:**
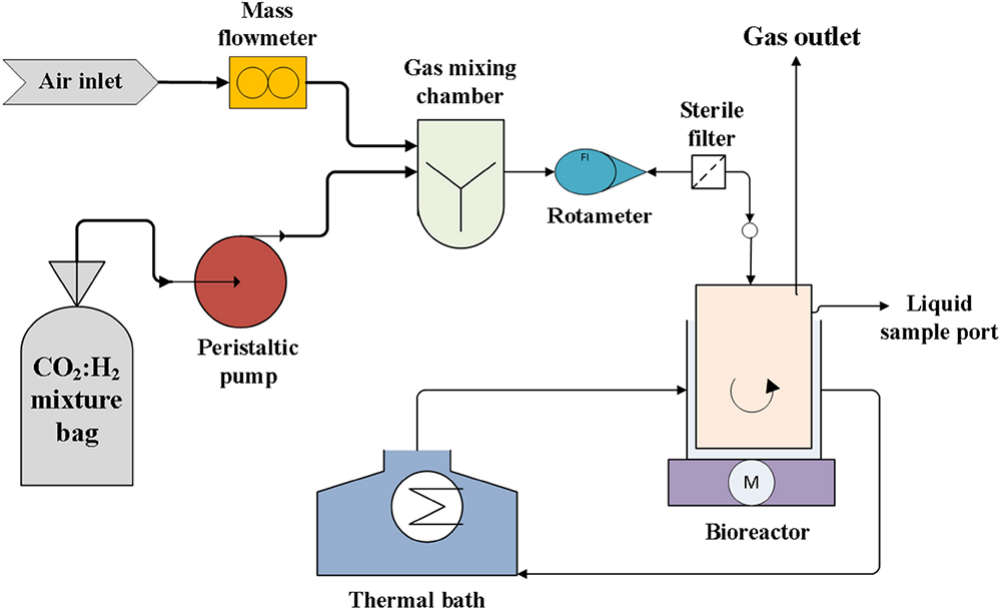
Schematic Overview of the Experimental Setup

A CO_2_–H_2_-Air mixture emission (concentrations shown in Table 1) was continuously fed to the STR via a stainless-steel porous diffuser (2 μm, Supelco, USA). This polluted air emission was obtained by mixing a 20% H_2_: 80% CO_2_ (v/v) stream with a continuous air flow. The CO_2_:H_2_ mixture was fed into the system via a peristaltic pump (Watson-Marlow 313D) from 50 L multifoil bags with polypropylene fitting (MediSense, The Netherlands) and homogenized in a mixing chamber with the continuous air flow, which was regulated by a mass flow controller (Aalborg, USA). The flow rate of the resulting polluted-air emission was regulated by calibrated rotameters (Aalborg, USA) prior entering the bioreactor.

**Table 1:** Cultivation and Operational Conditions Evaluated during H. marinus Growth

operational run	CO_2_ inlet (g m^–3^)	H_2_ inlet (g m^–3^)	O_2_ inlet (g m^–3^)	EBRT (min)	*T* (°C)	run duration (days)	dilution rate (d^–1^)
Experimental assay 1 (E1)							
R I	198.0 ± 7.3	31.5 ± 1.7	80.3 ± 2.3	60	25	26	0.05
R II	241.2 ± 7.1	38.1 ± 1.1	66.8 ± 1.6	120	25	26	50 mL replacement
							0.10
R III	229.2 ± 17.7	35.7 ± 2.4	67.6 ± 4.6	120	37	26	0.05
							0.05 + (biomass return)
R IV	244.0 ± 9.4	42.3 ± 2.3	70.0 ± 4.8	120	37	26	0.25
Experimental assay 2 (E2)							
R I	218.0 ± 4.3	41.5 ± 1.9	81.3 ± 3.3	120	37	26	0.25

To prevent contamination, air filters (0.22 μm) were placed in the inlet gas flow. Monitorization of CO_2_, O_2_ and H_2_ concentrations was conducted to determine CO_2_, O_2_ and H_2_ consumption by H. marinus. For this, gas samples were periodically taken from the sampling ports located at the inlet and outlet of the bioreactors using 250 μL gastight syringes (HAMILTON, Australia). Steady state conditions were achieved when the elimination capacity (H_2_-EC) deviated <10% from the mean for at least 5 days.

For all experimental runs, 10 mL of the liquid culture were collected daily with a 50 mL sterile syringe from a designated sampling port in the reactor to assess biomass dry weight, pH, and ectoine content, and this volume was replenished with 10 mL of fresh AMS. To assess Dissolved Inorganic Carbon (DIC) and Total Organic Carbon (TOC), 20 mL of the withdrawn culture broth were used. All the experimental assays and operational runs are shown in Table 1.

#### 2.3.1 Experimental Assay 1 (E1): Screening of Different Operational Parameters to Evaluate Ectoine Productivities and Bioreactor Stability

E1 consisted of four operational runs of 26 days-each. In Run I, the EBRT was maintained at 60 min by controlling the polluted-air mixture inlet flow rate at 16 mL min ^–1^. The reactor temperature was controlled at 25 °C. A dilution rate of 0.05 d^–1^ was applied once the biomass reached the stationary phase.

In Run II, the EBRT was increased to 120 min to enhance H_2_ mass transfer. This was done by decreasing the polluted air mixture inlet flow rate to 8 mL min^–1^. The temperature of the reactor was maintained at 25 °C. A replacement of 50 mL of fresh AMS 6% was implemented in the bioreactor once the biomass reached the exponential growth-phase in order to promote biomass growth and stabilize ectoine content. The dilution rate was then increased to 0.10 d^–1^ when the biomass and ectoine contents started to decay. This approach aimed to facilitate biomass renewal at the beginning of operation while keeping secondary metabolite levels low.

In Run III, the EBRT was maintained at 120 min but the reactor temperature was increased to 37 °C (optimum growth temperature of the strain) by means of a water bath (Huber, Spain) connected to a glass jacket surrounding the bioreactor. Initially, the dilution rate was set at 0.05 d^–1^ once the biomass achieved stationary growth. After 16 days of operation, biomass was returned to the bioreactor while maintaining the dilution rate. To this aim, 50 mL of the culture broth were withdrawn from the bioreactor daily, and the biomass pellet was returned to the bioreactor after centrifugation at 7000 rpm for 20 min (Eppendorf, Spain) following resuspension in 50 mL of fresh AMS 6%. This strategy avoided biomass wash out during the second stationary phase.

In Run IV, the EBRT, temperature and dilution rate were maintained at 120 min, 37 °C and 0.25 d^–1^ to promote higher biomass and ectoine productivity.

#### 2.3.2 Experimental Assay 2 (E2): Optimization of Ectoine Productivities and Bioreactor Stability

E2 was conducted for 26 days with the optimum culture and operational parameters assessed from E1: EBRT of 120 min, 37 °C and a dilution rate of 0.25 d^–1^ once the biomass reached the exponential growth phase. Consequently, 500 mL of the culture broth were withdrawn every 2 days from the bioreactor and replenished with fresh sterile AMS supplemented with 6% NaCl to obtain high productivities of biomass and ectoine.

### 2.4 Analytical Methods

#### 2.4.1 Ectoine Determination

The intracellular ectoine was extracted with 4 mL of cultivation broth following the method reported by Cantera et al.^22^ Ectoine concentrations were determined using HPLC-UV analysis with a 717 plus autosampler (Waters Alliance e2695, USA) coupled to a UV Dual λ Absorbance detector (Waters, USA) at 220 nm and 40 °C. The separation was carried out using an LC-18 AQ p C Supelcosil column and a C18 AQ + precolumn, both from Sigma-Aldrich (Spain). The mobile phase consisted of a phosphate buffer containing 0.8 mM K_2_HPO_4_·3H_2_O and 6.0 mM Na_2_HPO_4_·12H_2_O, maintained at 25 °C with a flow rate of 1 mL min^–1^. Ectoine quantification was performed with external standards of commercially available ectoine [(*S*)-β-2-methyl-1,4,5,6-tetrahydropyrimidine-4-carboxylic acid, 95% purity] (Sigma-Aldrich, Spain).

For ectoine quantification, external standards of commercially available ectoine [(*S*)-*b*-2-methyl-1,4,5,6-tetrahydro-pyrimidine-4-carboxylic acid, purity 95%] (Sigma-Aldrich, Spain) were employed. The specific intracellular ectoine content was calculated based on eq 1.

$$[\mathrm{Ect}\;\mathrm{SP}]\;(\frac{{\mathrm{mg}}_{\mathrm{ect}}}{g_{\mathrm{biomass}}})=\frac{\mathrm{mg}\;\mathrm{intra}-\mathrm{cellular}\;\mathrm{ectoine}}{g\;\mathrm{dry}\;\mathrm{weight}\;\mathrm{biomass}}$$ 1

#### 2.4.2 CO_2_ and H_2_ Monitorization

The concentrations of CO_2_ and H_2_ gases were analyzed using an Agilent 7890A GC-TCD (Agilent Technologies, USA) equipped with a CP Poraplot Q column (CP7554, 25 m × 0.53 μm × 20 μm). The temperatures of the oven, injector, and detector were maintained at 45, 150, and 200 °C, respectively. Helium was used as the carrier gas at a flow rate of 13.7 mL min^–1^.

The concentration of CO_2_ in the aqueous phase was determined as the concentration of dissolved inorganic carbon (DIC). The aqueous CO_2_ concentration was measured using a total organic carbon (TOC) analyzer TOC-VCSH (Shimadzu, Japan). Prior to TOC analysis, all samples were filtered through a 0.22 μm membrane and adjusted to pH 9 with 0.5 M NaOH (for DIC samples) or 0.5 M HCl (for TOC samples). The total CO_2_ content was calculated as the sum of the CO_2_ concentrations in both the gas and aqueous phases.

#### 2.4.3 Biomass Determination

Optical absorbance measurements at 600 nm (OD600) were conducted using a SPECTROstar Nano at 600 nm (BMG LABTECH, Germany). Dry biomass concentration was calculated as total suspended solids (TSS) according to Standard Methods.^28^ Volatile Suspended Solids (VSS) were not assessed as in a pure strain culture with no other organic sources present in the medium, VSS are equivalent to TSS. The doubling time (*G*) was calculated according to eq 2:

$$G\;(h)=\ln(2)*(\frac{\left(t_{2}-t_{1}\right)}{\ln(\frac{i_{2}}{i_{1}})})$$ 2

where, *G* is the generation time; *t*_1_ and *t*_2_, time 1 and time 2; *i*_1_ and *i*_2_, OD600 at time 1 and time 2.

The pH of the medium was determined using a SensIONTM + PH3 pH meter (HACH, Spain).

#### 2.4.4 Data Treatment

Removal efficiency (% RE) was calculated using daily CO_2_ and H_2_ concentration measurements as described in eq 3:

$$\mathrm{RE}\;(\%)=100\times (1-\left(\frac{C_{\mathrm{out}}}{C_{\mathrm{in}}}\right))$$ 3

Where, *C*_in_ and *C*_out_ are the inlet and outlet concentrations (g m^–3^), respectively, of each gas.

#### 2.4.5 Statistical Analysis

Statistical analyses were conducted using SPSS 26.0 (IBM, USA). Significant differences were assessed by ANOVA and post hoc analysis for multiple group comparisons considering homoscedasticity and heteroscedasticity depending on Levene test results. Differences were considered significant at *p* < 0.05.

## 3 Results and Discussion

### 3.1 Influence of Operational Conditions on Ectoine Production and CO_2_ and H_2_ Consumption (E1)

#### 3.1.1 Influence on Ectoine Productivity

Results of ectoine contents and ectoine productivities during E1 are shown in Figures 1 and 2. After inoculation with an active culture of H. marinus at 50 mg L^–1^ and pH 7.0 ± 0.2, a lag phase of 3 days was observed in Run I (Figure 1a). Biomass reached an exponential growth phase at day 4, with maximum biomass values of 100.0 ± 2.3 mg L^–1^ by day 9. Concomitantly, specific ectoine production showed the highest maximum content on day 4 with average values of 108.4 ± 8.9 mg_Ect_ g_biomass_^–1^, attributed to the initial hyperosmotic shock experienced by the cells during the exponential growth. From day 9 to 16, stationary growth was observed with similar biomass values, but ectoine contents decreased to nearly half of the initial levels (57.4 ± 3.9 mg_Ect_ g_biomass_^–1^). This significant decrease could be associated with ectoine reassimilation by the cells for metabolic purposes during the stationary phase.^29^ To encourage new biomass growth and therefore, increase ectoine contents, a dilution rate of 0.05 d^–1^ was applied on day 17. This adjustment aimed to favor biomass renewal and keep the cells in continuous exponential growth. Unexpectedly, this change negatively affected ectoine production with ectoine contents dropping to 22.1 ± 9.1 mg_Ect_ g_biomass_^–1^and productivities of 2.1 g_Ect_ m^–3^ d^–1^. Extracellular ectoine was previously examined during batch studies (data not shown), with no detectable levels observed. Additionally, a BLASTp analysis confirmed the absence of ectoine ABC membrane transporters (EhuABCD) and homologous proteins in H. marinus, indicating a lack of genomic capacity for extracellular ectoine transport. Therefore, the observed reduction in ectoine concentration is unlikely to be associated with its release into the extracellular environment. We attributed this outcome to cell-age potentially caused by the poor solubility of H_2_ which resulted in energy constrains for the biomass. Most likely, the metabolic state of these older cells was focused on cell maintenance, which together with the low availability of the energy source at an EBRT of 60 min prompted the degradation of the previously synthesized ectoine serving as an energy source.^29^

**Figure 1:**
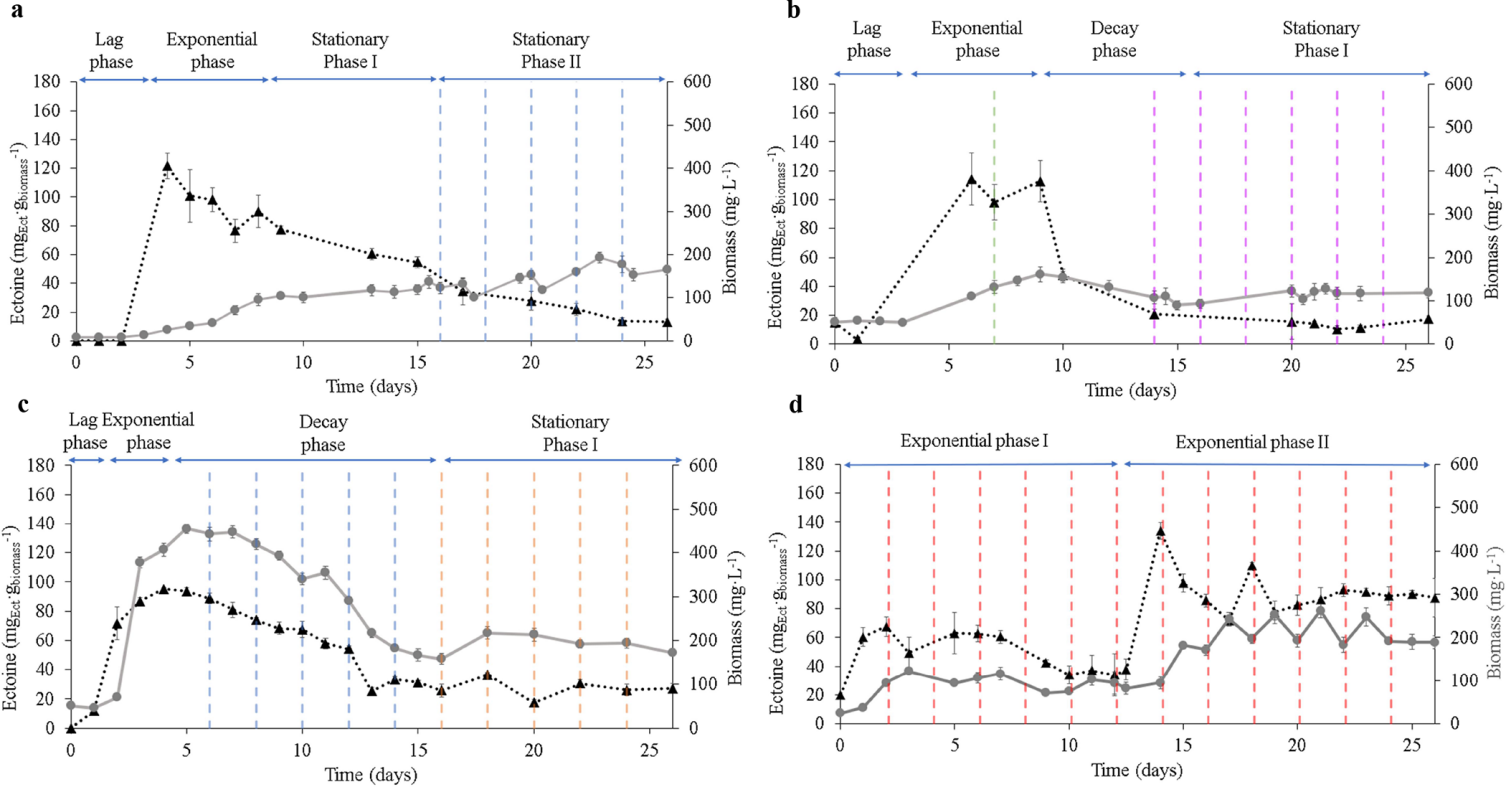
Specific ectoine contents and biomass production in (a) run I; (b) run II; (c) run III; and (d) run IV. Specific ectoine contents (▲ dark line) and biomass contents (● gray line). Application of dilution rates: (0.05 d^–1^, blue dashed line; 0.05 d^–1^ with biomass return, orange dashed line; 0.10 d^–1^, purple dashed line; 0.25 d^–1^, red dashed line). Replacement of 50 mL of fresh AMS 6% (green dash line). Data are presented as the average value ± SD of triplicate biological measurements.

**Figure 2:**
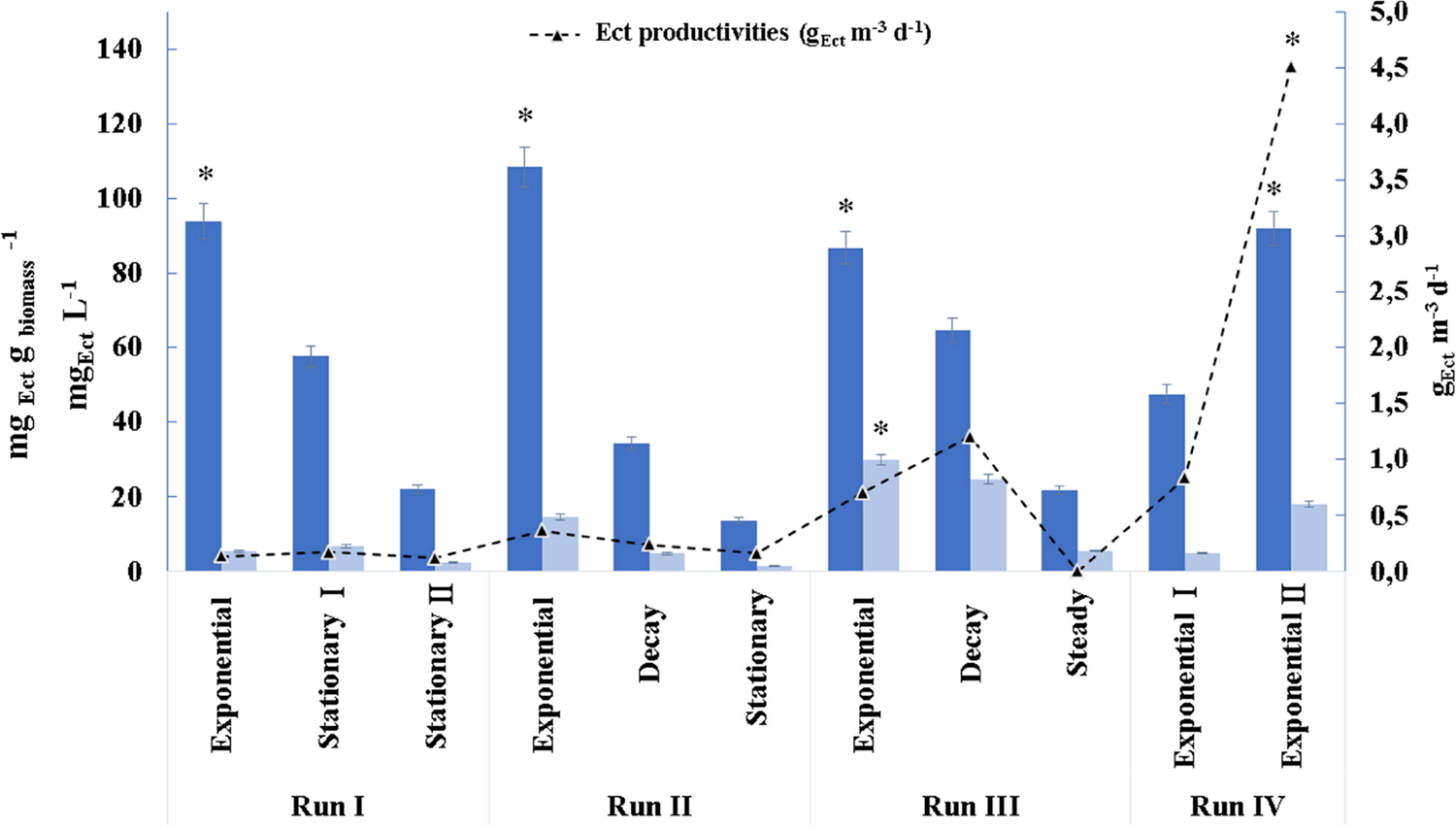
Average specific ectoine contents, ectoine concentrations, and ectoine productivities obtained during each run assessed in E1. Average specific ectoine contents (light blue bars), average ectoine concentrations (dark blue bars), and ectoine productivities (dash line). Data are presented as the average value ± SD of triplicate biological measurements. * Difference is significant at the *p* < 0.05 level between each biomass growth phase and run.

Therefore, in Run II (Figure 1b), the EBRT of the system was increased to 120 min, to enhance energy availability and ectoine production. A lag phase of 3 days was also shown in Run II. Exponential growth began on day 4. Biomass concentration peaked at 160.8 ± 16.7 mg L^–1^ on day 9, and maximum ectoine contents reached an average of 114.4 ± 18.2 mg_Ect_ g_biomass_^–1^ on day 4. During the exponential growth phase, a replacement of 50 mL was implemented in the bioreactor, which effectively promoted biomass growth and stabilized ectoine contents by renewing the biomass and removing toxic metabolites from the medium. However, by day 10, both biomass and ectoine contents began to decline, with ectoine contents falling to 20.7 ± 4.1 mg_Ect_ g_biomass_^–1^, likely due to cell age and the persistent accumulation of toxic metabolites. Studies have reported that chemolithoautotrophic bacteria accumulate small-molecule organic compounds (such as amino acids and organic acids) during CO_2_ assimilation if growth is not exponential and they are in a stationary metabolic state. These compounds repress CBB gene transcription, reducing the CO_2_ assimilation rate.^30^ This disruption may affect the overall energy balance in the cell, shifting its focus toward maintenance functions, thus reducing the production of precursors for ectoine biosynthesis and diminishing the cell’s capacity to synthesize ectoine. Consequently, a dilution rate of 0.10 d^–1^ was applied on day 14. This adjustment allowed the stabilization of ectoine contents and biomass concentrations for the remainder of the run. Nonetheless, it did not facilitate the recovery of biomass and ectoine concentrations to their initial levels.

Therefore, in Run III (Figure 1c), the EBRT of the system was maintained at 120 min to enhance energy availability and ectoine production. Moreover, the temperature was set at 37 °C to promote optimum cell growth. This change positively enhanced biomass growth (inoculated at 51 mg L^–1^, pH 6.9 ± 0.2) and reduced the lag phase to 2 days. Exponential growth was detected on day 3, concomitantly to ectoine production that reached by day 4 maximum values of 94.4 ± 1.1 mg_Ect_ g_biomass_^–1^. Here, biomass concentrations achieved levels three times higher than those in Run II due to the improved growth conditions. Despite these improvements, ectoine contents started to decay on day 6 (81.0 ± 7.8 mg_Ect_ g_biomass_^–1^) likely due to the accumulation of inhibitory metabolites, supported by a significant increment on TOC values from 63.2 ± 8.5 to 125.6 ± 16.8 mg L^–1^. In response, a dilution rate of 0.05 d^–1^ was implemented from this day to promote metabolites washout and cell renewal. Unexpectedly, this change did not result in higher ectoine levels, which fell further to 29.3 ± 5.6 mg_Ect_ g_biomass_^–1^ by day 13, nor did it affect biomass growth. On day 16, biomass retention was promoted by returning the biomass to the bioreactor to avoid cell washout, while maintaining the same dilution rate (0.05 d^–1^). This adjustment stabilized both ectoine production and biomass concentrations, however, the ectoine concentration represented only 5.3 ± 1.3 mg L^–1^. Although increasing the temperature reduced the lag phase and significantly boosted biomass content, excessive biomass accumulation and its subsequent recycling did not enhance ectoine levels.

To prevent the accumulation of toxic metabolites and maintain cell-age in exponential growth, in Run IV (Figure 1d), the same operational parameters (EBRT of 120 min, 37 °C) were maintained as in Run III, but dilution rate was increased to 0.25 d^–1^ once the biomass reached the exponential phase (day 3). During this phase, ectoine contents remained stable at 62.6 ± 11.0 mg_Ect_ g_biomass_^–1^, obtaining ectoine productivities of 0.8 g_Ect_ m^–3^ d^–1^. However, biomass and ectoine levels were significantly lower than in previous runs, this was most likely due to the pH variation as a result of the high dilution rate and the lack of pH control (pH reached values of 8.2). To address this, the reactor pH was adjusted on day 12 by replacing 500 mL of fresh AMS 6% which had been preadjusted to 7.0 ± 0.2 in an abiotic reactor with continuous CO_2_ and H_2_ supply, at concentrations of 244.0 ± 9.4 and 42.3 ± 2.3 g m^–3^, respectively (exponential phase I, Figure 1d). This adjustment improved the exponential growth phase, significantly increasing biomass concentration and ectoine levels to 247.8 ± 25.8 mg L^–1^ and 89.0 ± 14.5 mg_Ect_ g_biomass_^–1^, respectively. The maximum specific ectoine content was observed on day 13, reaching 134.0 ± 5.8 mg_Ect_ g_biomass_^–1^, together with average ectoine productivities of 4.5 ± 0.2 g_Ect_ m^–3^ d^–1^. Compared to previous runs, three times higher ectoine productivities were obtained, which corresponded to average specific ectoine contents of 8% of the total biomass dry weight (exponential phase II, Figure 1d). These values are comparable with the ones reported by Cantera et al.,^27^ showing similar average specific ectoine contents of 72.2 ± 10.7 mg_Ect_ g_biomass_^–1^ for H. marinus growing at 6% NaCl in batch studies.

#### 3.1.2 Influence on CO_2_ and H_2_ Consumption

In Table 2, the RE (%) of CO_2_, H_2_ and O_2_ were compared across the different experimental runs tested in E1.

**Table 2:** RE (%) of CO_2_, H_2_, and O_2_ by H. marinus during Each Operational Run Evaluated in E1

					average RE (%)		
Experimental assay 1 (E1-Run)	biomass growth phase	*T* (°C)	dilution rate (d^–1^)	pH	RE-CO_2_	RE-H_2_	RE-O_2_
E1-Run I	exponential	25	0	6.8 ± 0.1	3.0 ± 1.1^a^	6.7 ± 1.8^a^	13.7 ± 3.4^a^
	stationary I	25	0	6.6 ± 0.1	2.5 ± 1.2^a^	14.2 ± 3.6^b^	15.9 ± 2.5^a^
	stationary II	25	0.05	6.6 ± 0.1	3.5 ± 1.3^a^	14.7 ± 3.2^b^	10.8 ± 4.5^a^
E1-Run II	exponential	25	50 mL replacement	6.6 ± 0.1	3.2 ± 0.3^a^	18.2 ± 2.0^b^	10.2 ± 2.1^a^
	decay	25	0.1	7.0 ± 0.3	2.0 ± 0.9^a^	19.1 ± 4.9^b^	9.2 ± 1.8^a^
	stationary	25	0.1	6.6 ± 0.1	2.3 ± 1.3^a^	12.4 ± 2.8^b^	10.2 ± 1.6^a^
E1-Run III	exponential	37	0.05	6.6 ± 0.1	3.8 ± 1.6^a^	18.8 ± 1.2^b^	40.1 ± 5.9^b^
	decay	37	0.05 (biomass back)	6.7 ± 0.3	3.5 ± 2.5^a^	10.8 ± 3.9^b^	21.4 ± 3.6^c^
	stationary	37	0.05 (biomass back)	6.7 ± 0.1	8.7 ± 4.3^b^	10.6 ± 2.0^b^	19.9 ± 2.2^c^
E1-Run IV	exponential I	37	0.25	6.7 ± 0.4	6.8 ± 2.1^b^	17.8 ± 5.6^b^	27.3 ± 3.2^c^
	exponential II	37	0.25	6.6 ± 0.1	8.9 ± 1.6^b^	24.4 ± 2.3^c^	40.6 ± 5.2^b^

^a^Values with different letters for each parameter are statistically different at *p* < 0.05 for each biomass growth phase and run. Data are presented as the average value ± SD of triplicate technical measurements.

Generally, similar RE-CO_2_ values were achieved during the exponential growth phase of Runs I, II, and III (approximately 3%). These results are consistent with those reported by Cantera et al.^27^ for the H. marinus strain at 6% NaCl (2.3 ± 0.2%). In contrast, RE-H_2_ increased significantly in Runs II, III, and IV, likely due to the increase in EBRT to 120 min, which enhanced the solubility of H_2_ (almost ∼2 folds). However, RE-H_2_ did not show any significant increase in Run III (18%) despite the substantial rise in biomass content. This is likely attributed to limitations in dissolved oxygen (RE-O_2_ of 40.1 ± 5.9%) caused by the higher biomass levels observed during this run, which could potentially affect the stoichiometric H_2_ uptake. Furthermore, the accumulation of biomass may have led to nutrients depletion, such as nitrogen, sulfur, phosphorus, potassium, and manganese, which are critical for bacterial metabolism.^31^ Additionally, it could also cause the depletion of Fe^2+^, which is crucial for H. marinus membrane-bound respiratory [NiFe]-hydrogenases (MBH) activity and the function of electron transfer within the respiratory chain.^32^

During the decay phase of Run II and III, RE-CO_2_% become unstable (2.0 ± 0.9 and 3.5 ± 2.5, respectively), likely due to the accumulation of inhibitory compounds supported by TOC concentrations recorded during these stages (TOC 183.7 ± 11.4 mg L^–1^). The stationary and decay stages typically exhibited higher RE-H_2_ values. This can be attributed to the increased energy (H_2_) required by the biomass to produce 1 C-mol cell mass during the decay and stationary growth phases in contrast to the exponential growth phase, as shown in eqs 4 and 5.^33^

Exponential growth phase:

$${\mathrm{CO}}_{2}+7.77H_{2}+2.87O_{2}+0.24{\mathrm{NH}}_{3}\to {\mathrm{CH}}_{1.68}O_{0.46}N_{0.24}+7.28H_{2}O$$ 4

Stationary and decay phase:

$${\mathrm{CO}}_{2}+13.48H_{2}+5.72O_{2}+0.24{\mathrm{NH}}_{3}\to {\mathrm{CH}}_{1.68}O_{0.46}N_{0.24}+12.98H_{2}O$$ 5

The H_2_ consumption is one of the critical factors that determine the economic feasibility of CO_2_ fixation when using hydrogen-oxidizing bacteria for the production of ectoine.^33^ Minimizing the use of H_2_ while maximizing CO_2_ consumption to produce high yields of biomass would be the most favorable approach. Thus, achieving stationary and decay phases in the STR are neither desirable nor economically attractive. A previous techno-economic analysis study estimated that ectoine production costs using methane as the carbon and energy source, range from 158 to 231 €/kg-ectoine, significantly below current ectoine manufactury values.^34^ However, the considerable CO_2_ emissions associated with methanotrophic growth challenge the eco-friendly claims of CH_4_-based bioprocesses.^24^ In contrast, our study suggests that using hydrogen-oxidizing bacteria could mitigate this issue, while also enables the integration of existing industrial gas streams containing CO_2_ and H_2_ as feedstock, such as pretreated syngas from biomass or coal gasification or waste gas streams from sources such as natural gas-fired power plants.

During Run IV, the removal of both H_2_ and CO_2_ reached significantly higher values, particularly during the second exponential growth phase (8.9 ± 1.6 and 24.4 ± 2.3%, respectively). This improvement is attributed to maintaining the biomass in a continuous exponential growth phase by applying a higher dilution rate. Here, the increased dilution rate facilitated the removal of toxic metabolites (with TOC levels of 65 mg L^–1^) and enhanced CO_2_ consumption. H. marinus is identified as an obligate chemolithoautotrophic bacterium capable of fixing CO_2_ as its sole carbon source via the Calvin-Benson-Bassham (CBB) cycle.^35^ Due to RuBisCO’s bicatalytic nature and limited carboxylation efficiency, CO_2_ utilization by autotrophs is typically not efficient. However, previous studies showed that when biomass enters the exponential growth phase, it promotes CBB gene transcription and the synthesis of cellular materials, thereby boosting cell growth and CO_2_ fixation yields.^36,37^ In this scenario, the results obtained from E1 showed that an EBRT of 120 min enhanced gas transfer efficiency and improved the removal of H_2_ and O_2_ within the bioreactor. Maintaining the optimal growth temperature at 37 °C resulted in a shorter doubling time, thereby promoting optimal growth and major resilience of the biomass. Additionally, employing a higher dilution rate of 0.25 d^–1^ during the initial exponential growth phase facilitated the removal of older biomass, avoided the accumulation of toxic metabolites and promoted CO_2_ fixation. Consequently, these findings elucidated the optimal strategies for continuous STR operation with H. marinus, leading to the selected parameters for conducting assay E2.

### 3.2 Experimental Assay 2 (E2): Optimization of Ectoine Productivities and Bioreactor Stability

The optimum culture and operational parameters to enhance the production of ectoine under continuous operation with H. marinus, were applied in E2 (Figure 3a). Biomass growth began immediately on day 1 (182.3 ± 11.5 mg L^–1^), exhibiting a negligible lag phase. Concomitantly, the maximum specific ectoine concentration was reached, achieving 134.0 ± 6.3 mg_Ect_ g_biomass_^–1^ (13.4% of the total dry weight), likely due to the initial hyperosmotic shock experienced by the cells. A dilution rate of 0.25 d^–1^ was implemented once the biomass entered the exponential growth phase (day 2), until the end of the experiment. As expected, applying the dilution rate during this stage did not result in a disruption of the biomass’s exponential growth, facilitating the washout of secondary metabolites and old biomass from the bioreactor, as supported by TOC values of 61–73 mg L^–1^. By day 8, the specific ectoine content reached a steady-state value, averaging 87.1 ± 10.6 mg_Ect_ g_biomass_^–1^ (9% of the total dry weight), comparable to those obtained in E1-Run IV. On day 16, biomass levels started to increase significantly, reaching a maximum of 353.5 ± 10.1 mg L^–1^ by day 24. This increase was likely driven by the ongoing biomass washout, which progressively boosted the active exponential cell population within the reactor. The specific ectoine content did not show a significant decline at this stage (average 78.9 ± 10.7 mg_Ect_ g_biomass_^–1^), considering that ectoine levels could be influenced by cellular adaptation. Consequently, applying the optimal parameters for ectoine production in the STR with H. marinus resulted in average ectoine productivities of 5.0 ± 0.3 g_Ect_ m^–3^ d^–1^. On the other hand, RE % for CO_2_ and H_2_ at the onset of the experiment (from day 1 to 15) were comparable to those recorded in E1-Run IV (7.1 ± 3.7% and 24.1 ± 4.4%, respectively). From day 16, both removal rates progressively increased over time, eventually doubling their initial values, with peak RE of 12.1% for CO_2_ and 49.7% for H_2_ by day 22, which corresponded with the significant increase in biomass content (Figure 3b). Although CO_2_ removal was significantly higher during E2 in comparison with the RE-CO_2_ obtained in E1, RE-CO_2_ remained limited, likely due to growth limitations caused by insufficient H_2_ transfer. This limitation resulted in reduced biomass growth rates and CO_2_ fixation efficiency. To improve CO_2_ uptake, several strategies could be explored such as scaling up the process in high mass-transfer bioreactors enhanced by internal gas recirculation systems (e.g., Taylor flow or gas bubble column reactors).^38,39^ Additionally, increasing the pH to the maximum tolerated by H. marinus growth (pH 8.5) could promote the capture of CO_2_ in the form of carbonates in the liquid phase.

**Figure 3:**
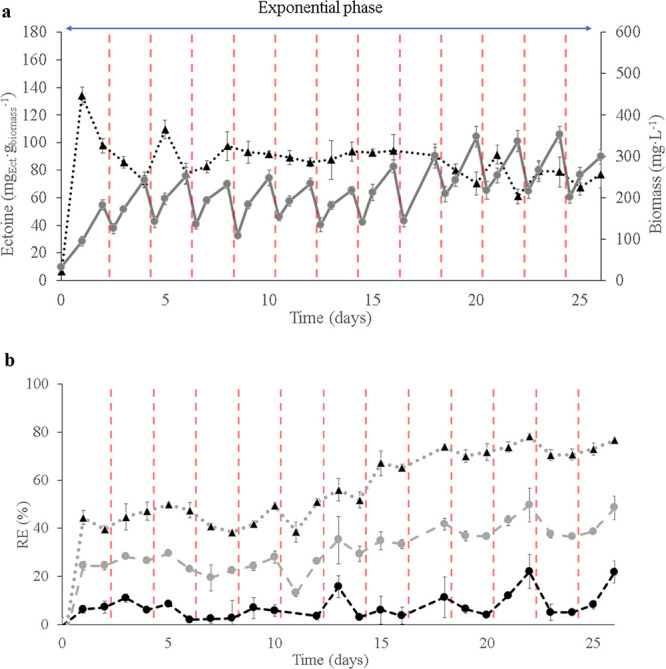
(a) Specific ectoine contents and biomass contents obtained during E2: Specific ectoine contents (▲ dark line) and biomass contents (● gray line). (b) Removal efficiencies (%) obtained during E2: RE-O_2_ (▲ gray line), RE-H_2_ (● gray line), RE-CO_2_ (● dark line). Application of dilution rate 0.25 d^–1^, red dashed line. Data are presented as the average value ± SD of triplicate biological measurements.

The ectoine yields obtained in this study were lower than those achieved with genetically modified microorganisms (GMOs). However, the use of GMOs lacks appeal in the cosmetic, pharmaceutical, and medical markets.^54^ The maximum specific ectoine content observed during E2 (134.0 ± 6.3 mg_Ect_ g_biomass_^–1^) exceeded values reported for methanotrophic bacteria utilizing methane or biogas, as well as hydrogenotrophic bacteria (Table 3). Moreover, this result is also comparable to those of heterotrophic strains, such as Brevibacterium epidermis DSM 20659 (160 mg_Ect_ g_biomass_^–1^) or with the industrial ectoine producer Halomonas elongata (100–180 mg_Ect_ g_biomass_^–1^) fed with high quality carbon sources, which reduces its cost-effectiveness.^44^

**Table 3:** Ectoine Production by Different Microorganisms

ectoine production				
microorganism	substrate	production yield (mg g_biomass_^–1^)	NaCl(M)	ref.
natural halophilic ectoine producers				
Hydrogenovibrio marinus DSM 11271	CO_2_ & H_2_	134	1.0	this study
Hydrogenovibrio marinus DSM 11271	CO_2_ & H_2_	85	1.0	^27^
Rhodococcus opacus DSM 43205	CO_2_ & H_2_	25	1.0	^27^
methanotrophic consortium	CH_4_	37	1.0	^40^
*Methylotuvimicrobium alcaliphilum* 20Z/DSM19304	CH_4_	75	1.0	^21^
*Methylotuvimicrobium alcaliphilum* 20Z/DSM19304	CH_4_	109	1.0	^22^
enrichment from marine coast sediment (Methylobacter marinus/whittenbury)	CH_4_	51	0.8	^41^
haloalkaliphilic consortium	biogas	57	1.5	^42^
methanotrophic consortium	biogas	79	1.0	^38^
methanotrophic consortium	biogas	94	1.0	^23^
Chromohalobacter salexigens DSM3043	glucose	540	1.8	^43^
Halomonas elongata BK-AG25	glucose	100–180	2.6	^44^
Brevibacterium epidermis DSM 20659	sodium glutamate and yeast extract	160	1.0	^45^
Halomonas elongata DSM2581	glucose	1365^a^	2.6	^46^
Halomonas salina DSM5928	sodium glutamate	358^a^	0.5^b^	^47^
Halomonas salina BCRC17875	sodium glutamate and yeast extract	14 (g L^–1^)^a^	2.0	^48^
genetically modified ectoine producers				
Halomonas hydrothermalis Y2	monosodium glutamate	765	1.1	^49^
*Corynebacterium glutamicum* ectABC^opt^	glucose and molasses	700	0.03	^50^
E. coli BW25113 (*pBAD-ectABC*)	aspartate, glycerol and glucose	4048^a^	0.5	^51^
E. coli DH5α (pASK_ectABCD_m_)	glycerol	2900^a^	0.01	^52^
*Methylotuvimicrobium alcaliphilum* 20ZDP2	CH_4_	110	1	^53^

a Excreted to the medium.

b Phosphate, citrate, and sulfate salts were also included.

This study represents the first proof of coupling ectoine production with the continuous abatement of CO_2_ using the strain Hydrogenovibrio marinus. Additionally, the results of this study provided new insights into the optimal operating conditions to improve ectoine productivity and biomass resilience. The EBRT was found to be crucial in process performance, primarily impacting the gas–liquid transfer of H_2_ and subsequently, the availability of H_2_ for hydrogenotrophic growth. Increasing the EBRT to 120 min significantly improved H_2_ solubility, resulting in a nearly 2-fold increase in RE-H_2_. Besides, maintaining the optimal growth temperature at 37 °C reduced the biomass lag phase to 2 days, thereby promoting optimal growth and improving biomass resilience. Implementing a dilution rate of 0.25 d^–1^ during the early exponential growth phase showed to be essential to remove aging biomass during cultivation in STR, preventing the accumulation of inhibitory metabolites and significantly increasing both biomass concentration and ectoine levels. By combining these operational parameters, we achieved maximum specific ectoine contents of 13.4% of the total biomass dry weight and average ectoine productivities of 5.0 ± 0.3 g_Ect_ m^–3^ d^–1^. The ectoine production values achieved in this study are either higher or similar and exhibit greater adaptability compared to those achieved using C_1_ sources, such as methane or biogas.

In this context, the long-term production of ectoine from CO_2_ and H_2_ by hydrogenotrophic bacteria represents a pioneering approach for advancing scale-up technology within the framework of next-generation biorefineries. Likewise, it highlights the need for further research to advance the implementation of CO_2_-based biorefineries for ectoine production. Moreover, the efficiency of this process can be significantly improved through laboratory-driven adaptation, the strategic use of engineered cocultures, and the integration of advanced bioreactors.
